# Elevating lived experience in overdose prevention: a community case study of Arizona’s overdose data to action community advisory board

**DOI:** 10.3389/fpubh.2025.1735257

**Published:** 2026-01-06

**Authors:** Martín F. Celaya, Devina Wadhera, Khenyan Wilcox, Dominic Orso

**Affiliations:** 1 Arizona Department of Health Services, Phoenix, AZ, United States; 2 Mel and Enid Zuckerman College of Public Health, University of Arizona, Tucson, AZ, United States

**Keywords:** advisory boards, community, community participatory evaluation, drug overdose, people with lived and living experience

## Abstract

**Introduction:**

Drug overdose remains a leading cause of preventable death in the United States, with persistent inequities among populations that often face stigma and barriers to care. Traditional evaluation models often exclude people with lived and living experience (PWLE) of substance use from meaningful decision-making.

**Methods:**

To address this, the Arizona Department of Health Services (ADHS) implemented a community-based participatory evaluation model within its Overdose Data to Action in States (OD2A-S) program by establishing a Community Advisory Board (CAB). The CAB was designed to integrate lived experience into evaluation planning and interpretation, identify community-driven priorities, and guide program improvement. Members were recruited statewide through a nomination process and compensated. A licensed clinical social worker facilitated meetings using trauma-informed and healing-centered approaches. Participatory methods such as journey mapping and logic model development enabled members to translate personal and community experiences into actionable evaluation questions.

**Results:**

Between August 2024 and August 2025, the CAB met quarterly to prioritize evaluation of naloxone vending machines and peer navigation models. Members identified key barriers, including stigma, rural access, transportation, and lack of culturally responsive care, and provided input on equitable evaluation tools and communication strategies. A composition survey confirmed strong representation of individuals in recovery (86%) and people of color (88%), though rural and tribal, medically assisted treatment, and veteran perspectives remain limited or not represented.

**Discussion:**

Early lessons demonstrate that compensated, trauma-informed engagement fosters trust, accountability, and practical insights that strengthen evaluation quality and relevance. Challenges include administrative delays in payment, limited generalizability due to small membership, and the need for sustainable funding to expand representation.

**Conclusion:**

Arizona’s OD2A-S CAB provides a replicable model for integrating lived experience into state-level overdose prevention. By positioning PWLE as co-evaluators rather than participants, this model advances equity, builds system trust, and strengthens the translation of data into action.

## Introduction

The growing burden of drug overdose in the United States stresses an urgent need for responsive, data-driven, and community-centered systems of prevention. Nationally, drug overdoses remain a leading cause of injury-related mortality, with rising trends driven by synthetic opioids and polysubstance use (1). The crisis reflects deep systemic inequities, disproportionately affecting White, non-Hispanic, and American Indian/Alaska Native communities, and people experiencing poverty, incarceration, housing insecurity, or living in rural areas (2). Individuals with co-occurring mental health conditions, trauma histories, or limited access to culturally responsive care also face heightened risk and barriers to treatment (3). Surveillance and intervention delivery become more effective when combined with participatory approaches because involving community voices ensures that the system is accountable to the needs of the people it serves and delivers interventions that are culturally appropriate and relevant.

Community-based participatory research (CBPR) and other similar frameworks center prevention and intervention efforts by involving the community as co-designers and co-implementors of the intervention. Specifically, these approaches engage people with lived and living experience (PWLE) of substance use not only as intervention participants but as co-producers of knowledge and co-designers of program improvement efforts. Traditional program evaluation methods often assign a greater value to institutional or academic approaches, thereby inadvertently excluding the insights of those most affected by the systems being evaluated. In contrast, community-driven evaluations aim to prioritize community expertise throughout the process, defining questions, interpreting findings, and guiding implementation of recommendations. This approach has been shown to improve program reach, enhance implementation fidelity, and foster greater trust between public health systems and affected communities (4, 5).

The CDC’s Program Evaluation Framework provides a guiding structure for embedding participatory approaches into public health evaluation (6). The framework emphasizes stakeholder engagement, contextual understanding, and iterative learning as core principles of evaluation practice. Additionally, public health evaluation scholarship emphasizes that program improvement requires feedback loops between implementers, evaluators, and affected populations (7, 8). Translating these principles into overdose prevention systems requires intentional strategies to ensure that lived experience is integrated not only in qualitative assessments but also in system-level decision-making, policy refinement, and utilization of evaluation findings. Participatory evaluation efforts within drug overdose prevention contexts illustrate the potential of such integration. Co-design processes between researchers and people who use drugs have been shown to improve the applicability of overdose prevention materials and implementation strategies (9–11). Similarly, participatory approaches engaging youth with lived experience of substance use highlight the value of relational ethics, shared decision-making, and sustained mentorship for systems learning (11). These findings collectively suggest that engagement does not just occur once but is a sustained relationship built on mutual learning, transparency, and shared accountability.

Recently published literature on participatory and lived-experience–informed evaluation models reinforces the need for systematic integration of community voices within state and local public health programs. Integrating community voices into evaluations can enrich the process by bridging the research-to-practice gap, strengthening partnerships, highlighting local priorities and realities, and uncovering gaps in assessment. Moreover, community voices, especially those from marginalized groups, can elevate and support the need to understand and address health disparities, promoting more equity-focused health initiatives. Excluding community voices in evaluations can risk creating programs that are less relevant, less applicable, and less effective in identifying and assessing true needs, assets, and gaps in care. In addition, studies demonstrate that engaging individuals with lived experience contributes to greater relevance and applicability of research findings, particularly in areas such as mental health and substance use, where social and contextual determinants play a major role (9, 12). However, engagement is often limited, fragmented, and confined with limited influence on evaluation design, interpretation, or dissemination and mostly represent symbolic consultation. Research led by people who use drugs challenges traditional systems that establish hierarchies of expertise by centering lived experience as a legitimate and essential form of evidence (13, 14).

Nonetheless, there are several considerations that need to be addressed and well thought out when planning to engage communities as co-creators and co-evaluators of interventions. The ethical and operational dimensions of community-based evaluation have been further explored by Souleymanov et al., who identified recurring challenges in compensation, representation, and institutional support when conducting CBPR with people who use drugs (14). Within overdose prevention, the need for structures such as community advisory boards is acute: state and local health departments manage large portfolios of surveillance, prevention, and response activities under programs like the Centers for Disease Control and Prevention’s Overdose Data to Action (OD2A) initiative, yet mechanisms for integrating community feedback into evaluation and system design remain limited.

Despite growing attention to participatory models, few state-level examples exist that systematically document the processes, outcomes, and lessons learned from integrating lived experience into overdose prevention evaluation. The present community case study addresses this gap by describing the formation, structure, and function of Arizona’s Overdose Data to Action in States (OD2A-S) Community Advisory Board (CAB). In CBPR, community advisory boards (CAB) provide structure and foundational support by guiding the research activities and represent the voices of the communities they serve. Community advisory boards consist of members who share common experiences, interests, or backgrounds of the community of interest. Thus, these shared experiences allow CABs to directly represent the community’s perceptions, preferences, and needs, thereby helping to shape the research agenda, content, and processes into one that is appropriate, relevant, and acceptable to the community.

It demonstrates how community perspectives have been embedded into evaluation and decision-making processes, how those perspectives have the potential to shape program adaptations, and the implications these experiences hold for future public health system design.

## Context/setting

**Figure 1:**
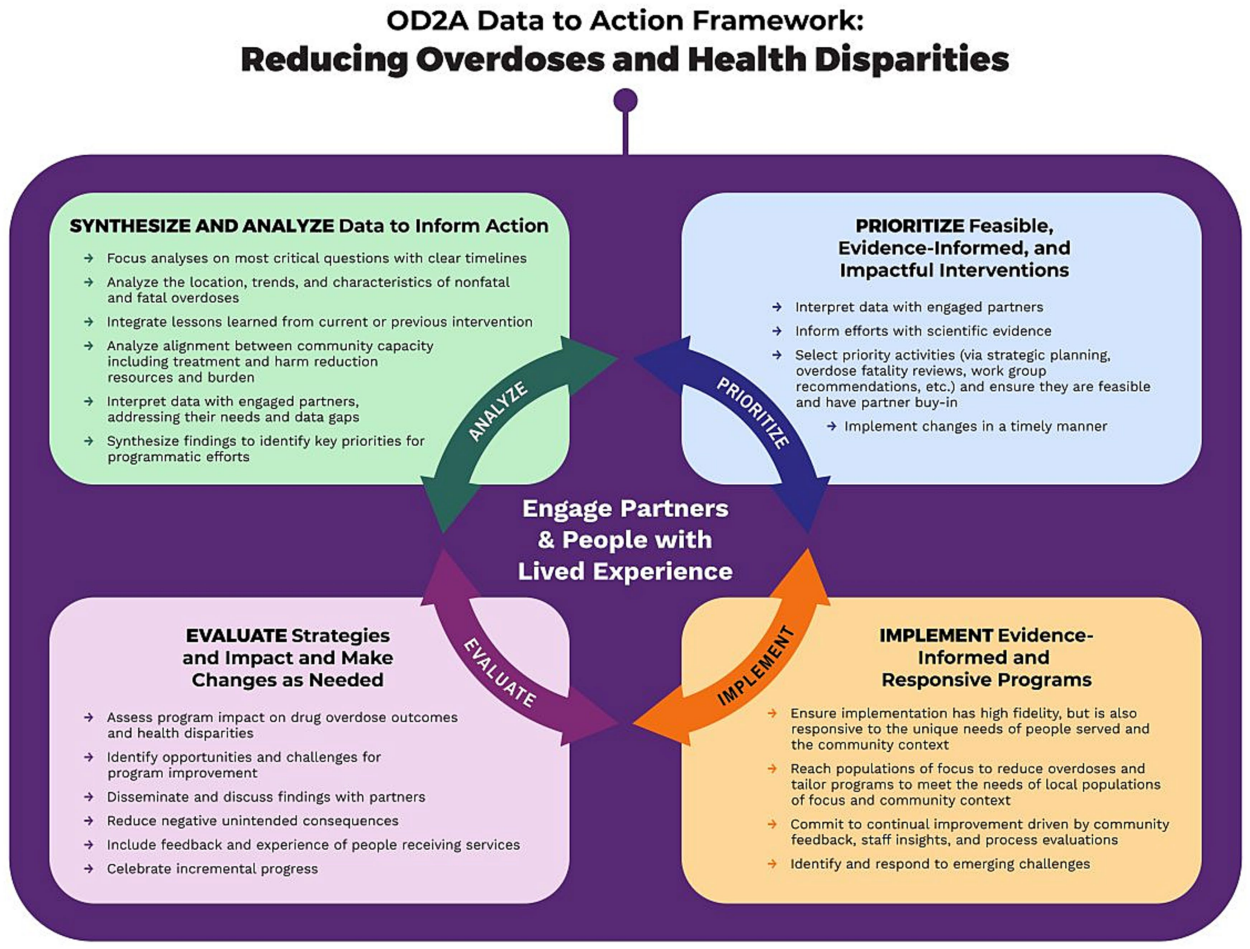
CDC OD2A data to action framework.

CDC launched the Overdose Data to Action (OD2A) initiative, an integrated public health strategy that emphasizes data to action as a guiding framework for addressing overdose prevention. This framework supports state and local jurisdictions in using surveillance and evaluation data to inform, implement, and assess comprehensive overdose prevention strategies while centering engaging partners and people with lived experience (Figure 1) (15).

**Figure 2:**
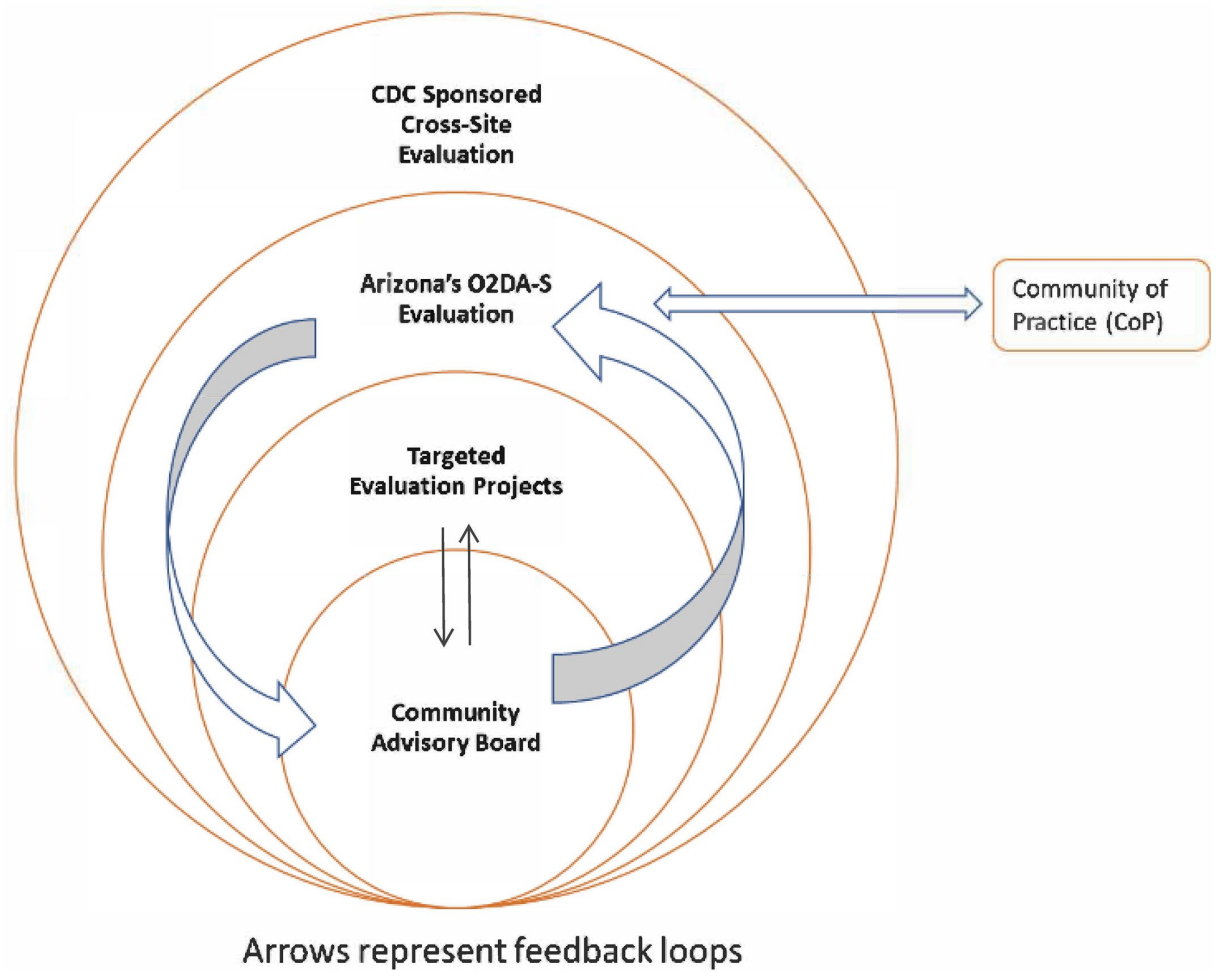
Arizona’s OD2A-S evaluation diagram.

Arizona’s OD2A-S (Overdose Data to Action in States) evaluation builds upon the CDC’s Data to Action framework by emphasizing the inclusion of community voices at every stage of the evaluation process. The Arizona Department of Health Services (ADHS) designed a participatory evaluation model to ensure that program findings reflect the experiences of PWUDs (People who use Drugs), their families, and community-based partners. The evaluation is planned for 5 years of the CDC Cooperative Agreement with ADHS (2022–2027). The approach integrates community-based participatory evaluation (CBPE) methods into a multi-level structure that links local community engagement to state and national evaluation outcomes. The proposed evaluation is complex and multilevel, encompassing a CDC-sponsored cross-site evaluation, a state-specific OD2A-S evaluation, targeted evaluation projects focused on individual interventions, and a Community Advisory Board (CAB) serving as the foundation for participatory decision-making (Figure 2). The CAB provides feedback primarily to the statewide OD2A-S evaluation, and CAB insights are incorporated into targeted evaluation projects when the focus of those projects aligns with members’ lived experiences or community-identified priorities. This structure ensures that Arizona’s evaluation aligns with CDC’s national priorities while centering the voices of those most impacted by overdose. Arizona’s participation in the community of practice (CoP) ensures that Arizona’s evaluation uses best practices for evaluation and community engagement through shared learning with other federally funded sites.

The CAB was formally launched with a virtual kick-off meeting on 28 August 2024, beginning with three members from the community and a facilitator who is a Licensed Clinical Social Worker (LCSW). The LCSW was intentionally selected because of extensive experience as a counselor and behavioral health therapist with expertise in group counseling, family therapy, case management, and support services. Including an LSCW ensured that CAB discussions occur within a trauma-informed and healing-centered environment. CAB members have personal histories that include substance use, incarceration, loss, or systemic stigma; the LCSW’s facilitation provides emotional safety, helps prevent re-traumatization, and fosters a supportive space where all members feel valued and heard. This approach strengthens group cohesion and reinforces trust between community members and the evaluation team. In addition to clinical and trauma-informed expertise, the LCSW had prior experience facilitating community groups and working with lived-experience populations in participatory settings.

To coordinate the CAB’s activities, we established a multidisciplinary support team composed of the following: two co-evaluators for the OD2A grant, who oversee the evaluation design and ensures alignment with CDC’s national framework; a graduate student intern, who provides logistical coordination, data synthesis, and technical assistance; the health program manager overseeing OD2A prevention strategies, who ensures integration between evaluation priorities and program implementation; and (4) the LCSW, who co-facilitates CAB meetings using trauma-informed and culturally responsive practices.

Recruitment for the CAB is ongoing and was conducted through a statewide nomination form inviting overdose prevention and treatment partners to confidentially nominate individuals with lived experience. Nominators needed to attest to receiving consent from nominees prior to submitting their information. This approach led to five additional members joining the CAB, bringing the total to eight. Following recruitment, CAB members complete a CAB Composition Survey to evaluate board diversity and representation of communities most impacted by overdoses in the state. Results from this survey (*n* = 7) indicate that our CAB, had equal representation on sex, mostly identify as currently in recovery (86%) with 79 years of combined time in recovery (13.2 years mean, range: 4–24 years), were mostly between the ages of 35–54 years (71%), and currently resided in an urban setting (71%), with small representation from rural, tribal, and border communities (28%), have served as peer navigators or support specialists (71%), belong to racial/ethnic minority groups (88%), had a mean age of first drug use of 26 years, and began their drug use in their home (29%). A majority of members cited using marijuana (cannabis), alcohol, methamphetamine, hallucinogens, heroin, and cocaine as their most cited drug of choice, and 43% reported previous polysubstance use with stimulants and depressants. While members identified a variety of adverse and protective situations and activities they experienced during their time in active addiction, being a victim or witness of neighborhood violence, experiencing unemployment, participation in 12-step programs such as Narcotics Anonymous, incarceration or detention, experiences of discrimination or unfair treatment, community volunteering, and experiences of abuse (physical, sexual, emotional, or financial) were the most common experiences reported by at least 71% of members. A small number of members (≤ 43%) had received medically assisted treatment (MAT), received treatment for drug dependency, reported managing a mental illness, or resided in an Indian Reservation.

CAB members are compensated for their time and expertise through the Engaging Families and Young Adults Program (EFYAP). This program is managed by ADHS and serves as the mechanism to reimburse individuals with lived experience who contribute to program design, evaluation, and policy work. Through an EFYAP contractor, CAB members receive foundational training on participating in boards and committees and on concepts of health equity as part of their onboarding process. The members are also supported with administrative tools such as subcontractor agreements, W-9 forms, and timesheets. This model ensures that participation is ethical, compensated, and structured within existing state systems, while reinforcing the principle that lived experience is professional expertise. The CAB meets quarterly and its meetings are designed around participatory methods that promote reflection, collaboration, and co-learning. Meetings are typically held in person on the weekend, with the exception of the kick-off meeting, in a safe neutral location such as the Phoenix Public Library, where members can easily access the meeting space via public transportation and have access to local resources such as job training, Wi-Fi equipment, computers, classes, cultural events, and others. While in-person meetings posed geographical barriers and limitations to recruitment, we prioritized enrolling individuals who could attend in-person meetings, while also ensuring that they represented diverse experiences, demographics, and regions.

CAB members engage in activities such as logic model building, journey mapping to visualize recovery pathways, and quality improvement feedback sessions for statewide training materials and implementation, and evaluation planning sessions focused on overdose prevention and linkage-to-care strategies. For example, discussions in 2024 focused on naloxone vending machines and peer navigation models as priority interventions for targeted evaluations. Through this approach, CAB members act as co-evaluators, shaping evaluation design and questions, reviewing tools (e.g., interview guides, maps, and logic models), and interpreting findings to inform both local and statewide strategies. CAB members also receive training and education on effective overdose prevention strategies, laws and policies (e.g., Good Samaritan Laws), federal and state funding structures, and fundamental elements of evaluation.

## Programmatic details

The CAB is designed as both a participatory governance structure and an implementation partner for the evaluation. The CAB’s work is organized around three programmatic elements: (1) capacity building and onboarding, (2) participatory evaluation design, and (3) feedback and dissemination (Table 1). Each element reflects the principles of community-based participatory evaluation (CBPE) while aligning with CDC’s Data to Action framework for overdose prevention. Table 1 displays the CAB’s development and evaluation activities.

**Table 1:** Timeline of Arizona OD2A-S community advisory board development and evaluation activities (2022–2027).

Timeframe and milestone	Key activities completed or planned
Year 1: September 2023 to August 2024 *Planning and Infrastructure Development*	Formation of the ADHS evaluation team Develop scopes of work for the facilitator and CAB members Recruitment and contracting of the LCSW facilitator Establishment of administrative processes to compensate CAB members through the EFYAP Development of the community engagement framework and CAB onboarding materials Initial recruitment of CAB members through personal referrals
Year 1: February 2024 to August 2024 *Community Advisory Board Formation*	Dissemination of the CAB membership nomination form statewide to prevention and treatment partners for recruitment of additional members Administration of the CAB composition survey to assess the representation and diversity of membership Completion of onboarding activities for CAB members Orientation and training provided through EFYAP to CAB members Completion of a literature review on opioid overdose, overdose deaths, and interventions designed for prevention to inform CAB members
Year 2: September 2024 to December 2024 *Participatory Evaluation Design*	CAB is launched with three founding members and an LCSW facilitator Review of naloxone vending machine and peer navigation models as candidate evaluation topics Development of evaluation questions for each strategy, identification of evaluation stakeholders, and discussion on the utility of evaluation findings to improve public health practice CAB reviews and finalizes logic models for naloxone vending machines and peer navigation strategies
Year 2 – January to August 2025 *Implementation and Continuous Feedback*	Additional recruitment strategies were employed to recruit and onboard five additional members based on the composition survey findings CAB reviews draft data collection tools and metrics CAB develops ethical and trauma-informed evaluation procedures Journey maps are developed and reviewed in a group setting
Year 3 - September 2025 to August 2026 *Implementation and Continuous Feedback*	*Planned activities:* Review of journey map analysis for interpretation Data collection and analysis with CAB oversight CAB conducts a quarterly review of emerging findings and refines evaluation questions, as needed Members co-develop community dissemination products such as briefs, presentations, and infographics
Year 4–5 – September 2026–August 2027 *Ongoing Evaluation and Dissemination*	*Planned activities:* CAB conducts a quarterly review of emerging findings and refines evaluation questions, as needed CAB participates in data interpretation, lessons learned documentation, and dissemination CAB members present findings at conferences and participate in peer-to-peer learning CAB co-develops translational products based on evaluation findings Evaluation results are incorporated into future overdose prevention planning and priorities

ADHS, Arizona Department of Health Services; LCSW, Licensed Certified Social Worker; CAB, Community Advisory Board; EFYAP, Engaging Families and Young Adult Program.

### Capacity building and onboarding

The CAB began its first operational year (2024–2025) by focusing on capacity building among members. Through the EFYAP, CAB members were formally onboarded as compensated partners and completed foundational training modules on group participation, health equity, overdose epidemiology, overdose prevention strategies, state policies, and evaluation fundamentals. These trainings were facilitated by ADHS staff and contracted partners such as Diverseability Inc., which specializes in trauma-informed community engagement. Each member also signed an agreement that includes the project’s background, description of their participation (i.e., scope of work), compensation terms, and other additional items. These agreements are reviewed and renewed annually. Each CAB meeting begins with an opening activity and a review of group agreements (“Be present,” “Honor perspectives,” “Share the space,” “Respect confidentiality,” and “Be open to learning from others”). These agreements, developed collaboratively by members and facilitators, foster a sense of safety and mutual respect. The LCSW facilitator plays an essential role in reinforcing these norms and supporting members as they share personal experiences related to substance use, stigma, and recovery.

### Participatory evaluation design

By December 2024, CAB members had shifted from foundational training to participatory design activities that directly informed the targeted evaluation plan. During quarterly meetings, members reviewed Arizona’s overdose prevention strategies, literature reviews, and prioritized two focal areas for evaluation: naloxone vending machines, and peer navigation models. A naloxone vending machine initiative provides 24/7 anonymous access to naloxone, fentanyl test strips, condoms, gun locks, and other health safety prevention materials (16). CAB members discussed the accessibility and visibility of these machines, emphasizing the importance of placement, temperature regulation, and community awareness campaigns. Using materials (notepads, sticky notes, markers, and other supplies) and structured discussions, the CAB identified program maintenance considerations, community buy-in and awareness, privacy, and rural access as key implementation challenges for evaluation. Additional methodologies designed by the CAB will be employed in the remainder of 2025 and 2026, such as provider interviews, PWUD focus groups, and an audit of vending machine inventory data to produce recommendations for the successful implementation of these strategies.

The peer navigation model was identified as the second priority due to its potential to connect individuals who use drugs with recovery, housing, and behavioral health services (17). CAB members noted that peer navigators often serve as trusted entry points into care, particularly for people with histories of incarceration or stigma within healthcare systems. The CAB recommended exploring both quantitative and qualitative metrics, such as time to linkage, satisfaction with services, and perceived stigma, to evaluate program impact. Data collection methodologies have yet to be designed and implemented by CAB. These activities are planned for 2026.

In addition to the two focal areas, we have employed participatory methods such as journey mapping and logic model development to facilitate in-depth discussions. Logic models provide a framework for CBPR by guiding the research-community partnerships and developing sound, evidence-based programs by visually mapping the program’s resources, activities, and expected outcomes (6). Journey mapping is a novel, person-centered approach to visually illustrate an individual’s navigation through a complex system or experience and is increasingly being used in healthcare to better understand a patient’s experience. In the context of substance use, journey mapping may be used to visually narrate an individual’s experience from substance use through recovery so that nuances in these experiences can be captured more comprehensively (18). The journey mapping exercise helped members visualize the recovery continuum, identify structural barriers (e.g., transportation, stigma, lack of culturally responsive care), and address opportunities for intervention (18). Logic model sessions translated these insights into measurable outcomes and indicators that the evaluation team could incorporate into formal tools. These participatory exercises were facilitated by the LCSW and evaluation staff to ensure both emotional safety and methodological consistency.

### Feedback and dissemination

The CAB is also positioned as a key reviewer of evaluation products, data collection instruments, dissemination materials, and health promotion materials. Draft evaluation tools are vetted by the CAB prior to implementation to ensure readability, cultural relevance, and alignment with the interventions’ proposed outcomes. For example, the board provides input on survey and interview language used with PWUD and vending machines, helping organizations to ensure that terminology is person-centered and free of stigma. In addition, the CAB has been pivotal in recommending strategies for the dissemination of findings and the development of evaluation translational products to diverse audiences, including the promotion of vending machines in bus stops, developing partnerships with hotels for the placement of overdose reversal and educational materials, and saturation of naloxone educational materials in areas of high traffic for people who use drugs.

We plan to involve CAB members in data interpretation and dissemination during Years 4 to 5 (2026–2027) of the evaluation. Members will review preliminary findings, contribute to meaning-making sessions, and co-develop recommendations for program improvement. Dissemination will include community briefs, infographics, and presentations that highlight local perspectives alongside quantitative outcomes. CAB members will also be invited to present at national or state-level conferences as co-authors or co-presenters. We believe this will further strengthen their role as knowledge partners rather than subjects of evaluation.

The integration of these three elements appears to have supported a high attendance rate of 94%, although this observation has not yet been formally evaluated. This high level of participation underscores the value members place on this work, with several traveling from rural and tribal areas to attend the meetings. These elements operate in an iterative process that embodies the data-to-action principle by transforming lived experience into data-informed recommendations for program and policy change. A structured self-evaluation of CAB functioning, including member satisfaction, perceptions of voice and influence, and group capacity to navigate difficult discussions, is planned for later phases of the OD2A-S evaluation (Years 4–5).

## Discussion

The OD2A-S CAB demonstrates how CBPE can be operationalized within a state-level overdose prevention initiative (13). The CAB structure provided a consistent, reciprocal feedback mechanism between the ADHS, PWLEs, and partner organizations. Through meetings centered on journey mapping, naloxone distribution and training strategies, and peer navigator programs, participants were able to co-define meaningful evaluation questions and highlight key systemic barriers that directly influence overdose prevention outcomes. Through the integration of the Engaging Families and Young Adults Program (EFYAP) as a reimbursement mechanism, CAB members were equitably compensated for their time and expertise, allowing them to more fully participate in defining meaningful evaluation questions. This process also surfaced key systemic barriers, such as stigma, transportation challenges, and limited service availability in rural areas, that directly affect overdose prevention outcomes (19).

Most CAB members belonged to racial/ethnic minority groups, were in recovery, and served as peer navigators or recovery supporters. The diversity and representation of the members confirm alignment with its mission to center community voices most impacted by overdose. Lessons learned highlight that relationship-building, flexibility, and transparency are essential for sustaining engagement. The CAB’s emphasis on group agreements, respect, presence, confidentiality, and shared voice, along with the utilization of an LCSW-trained facilitator, created a psychologically safe space that allowed authentic conversations, inquisitive curiosity, and critical dialogue (12–14). This dynamic was particularly important in bridging technical evaluation methods (e.g., logic models) with lived experience narratives that contextualize overdose trends in Arizona’s communities. Additionally, aligning meeting activities with visual and participatory tools (journey mapping, annotation, whiteboard discussions) helped translate complex evaluation concepts into accessible formats, fostering mutual learning between ADHS and CAB members (5, 18, 20, 21).

For future applications, integrating CAB input earlier in project design, rather than post-implementation, could strengthen the evaluation and ensure interventions reflect community-led priorities from inception. The CAB model also highlights the importance of sustained funding for advisory mechanisms, especially for PWLE compensation and peer facilitator roles, to prevent tokenism and burnout. Finally, documenting and disseminating CAB outputs through accessible, community-facing channels (e.g., infographics, brief reports, social media posts, radio ads) will enhance the public’s accountability and trust, which can ultimately result in positive outcomes for communities most impacted by drug overdoses.

## Acknowledgment of any conceptual or methodological constraints

While the CAB evaluation process achieved meaningful engagement, several limitations should be acknowledged. First, representation constraints exist. Despite efforts to recruit diverse participants, certain groups, such as individuals currently using substances (especially opioids), veterans, individuals experiencing housing insecurity, tribal community members, rural residents, and patients of medically assisted treatment, remain underrepresented due to recruitment barriers including transportation limitations, stigma concerns, inconsistent or unstable contact information, conflicts with work or treatment schedules, among others.

Second, the evaluation scope was limited by the CAB’s advisory nature. Meetings primarily generated qualitative input and thematic priorities rather than quantifiable impact measures, which may limit the generalizability of findings across broader overdose prevention systems. Future cycles could strengthen this by integrating mixed-methods evaluation approaches and structured follow-up with CAB recommendations.

Third, compensation processes and administrative timelines occasionally delayed member participation and compensation, which may have affected participant commitment and engagement. Although the EFYAP mechanism improved inclusivity, bureaucratic requirements (e.g., sub-contracts, timesheets, and W-9 s) posed accessibility challenges for some participants unfamiliar with state systems. To address this, forms were printed and explained at CAB meetings to ensure that members had adequate support in understanding the requirements to support their compensation.

Fourth, costs to compensate CAB members can be a significant barrier to future CAB expansion efforts. CAB members from the Phoenix metropolitan area were compensated only for their time at a $60 U. S. dollar per hour rate, while individuals traveling from outside the Phoenix metropolitan area were compensated for their travel time and mileage in addition to their time participating in the CAB, as per the State of Arizona Accounting Manual’s guidelines. Additional funding would need to be identified in order to further grow the CAB to include members residing in rural, tribal, and other border communities throughout the state.

Lastly, meeting documentation and longitudinal tracking of CAB influence remain areas that need to be further defined. While each meeting produced tangible outcomes such as journey maps, feedback summaries, and evaluation question lists, there is limited evidence yet of how CAB insights directly altered OD2A program decisions or resource allocation. Capturing this impact will be vital for demonstrating the CAB’s value within CDC’s Data to Action framework. Nonetheless, the lessons learned from these constraints provide a critical foundation for strengthening future participatory evaluation efforts. They demonstrate that meaningful engagement requires not only inclusion but also sustained infrastructure, resources, administrative flexibility, and continuous feedback loops to ensure that community voices do more than inform programs.

## Conclusion

Arizona’s OD2A-S CAB illustrates how participatory evaluation has the potential to transform state-level overdose prevention efforts by positioning people with lived experience as equal partners in the production of public health knowledge. Through intentional structures for compensation, trauma-informed facilitation, and shared decision-making, the CAB has elevated community voices that are too often excluded from evaluation and policy processes. While logistical and representational challenges remain, the CAB’s early outcomes demonstrate that equitable engagement is both achievable and essential for responsive, data-to-action systems. As jurisdictions nationwide continue to address the overdose crisis, the Arizona model offers a replicable framework for embedding lived experience into evaluation design, implementation, and dissemination, advancing not only program effectiveness but also the broader goal of health equity and trust in public health systems.

## Data Availability

The original contributions presented in the study are included in the article/supplementary material, further inquiries can be directed to the corresponding author/s.
